# Self-amplification of oxidative stress with tumour microenvironment-activatable iron-doped nanoplatform for targeting hepatocellular carcinoma synergistic cascade therapy and diagnosis

**DOI:** 10.1186/s12951-021-01102-0

**Published:** 2021-11-08

**Authors:** Qiao-Mei Zhou, Yuan-Fei Lu, Jia-Ping Zhou, Xiao-Yan Yang, Xiao-Jie Wang, Jie-Ni Yu, Yong-Zhong Du, Ri-Sheng Yu

**Affiliations:** 1 Department of Radiology, Second Affiliated Hospital, School of Medicine, Zhejiang University, 88 Jiefang Road, Hangzhou, 310009 Zhejiang Province People’s Republic of China; 2 Institute of Pharmaceutics, College of Pharmaceutical Sciences, Zhejiang University, 866 Yuhangtang Road, Hangzhou, 310058 Zhejiang Province People’s Republic of China

**Keywords:** Chemodynamic therapy, Ferroptosis, Hepatocellular carcinoma, Magnetic resonance imaging, Organic/inorganic nanoplatform

## Abstract

**Background:**

Hepatocellular carcinoma is insensitive to many chemotherapeutic agents. Ferroptosis is a form of programmed cell death with a Fenton reaction mechanism. It converts endogenous hydrogen peroxide into highly toxic hydroxyl radicals, which inhibit hepatocellular carcinoma progression.

**Methods:**

The morphology, elemental composition, and tumour microenvironment responses of various organic/inorganic nanoplatforms were characterised by different analytical methods. Their in vivo and in vitro tumour-targeting efficacy and imaging capability were analysed by magnetic resonance imaging. Confocal microscopy, flow cytometry, and western blotting were used to investigate the therapeutic efficacy and mechanisms of complementary ferroptosis/apoptosis mediated by the nanoplatforms.

**Results:**

The nanoplatform consisted of a silica shell doped with iron and disulphide bonds and an etched core loaded with doxorubicin that generates hydrogen peroxide in situ and enhances ferroptosis. It relied upon transferrin for targeted drug delivery and could be activated by the tumour microenvironment. Glutathione-responsive biodegradability could operate synergistically with the therapeutic interaction between doxorubicin and iron and induce tumour cell death through complementary ferroptosis and apoptosis. The nanoplatform also has a superparamagnetic framework that could serve to guide and monitor treatment under T2-weighted magnetic resonance imaging.

**Conclusion:**

This rationally designed nanoplatform is expected to integrate cancer diagnosis, treatment, and monitoring and provide a novel clinical antitumour therapeutic strategy.

**Graphical Abstract:**

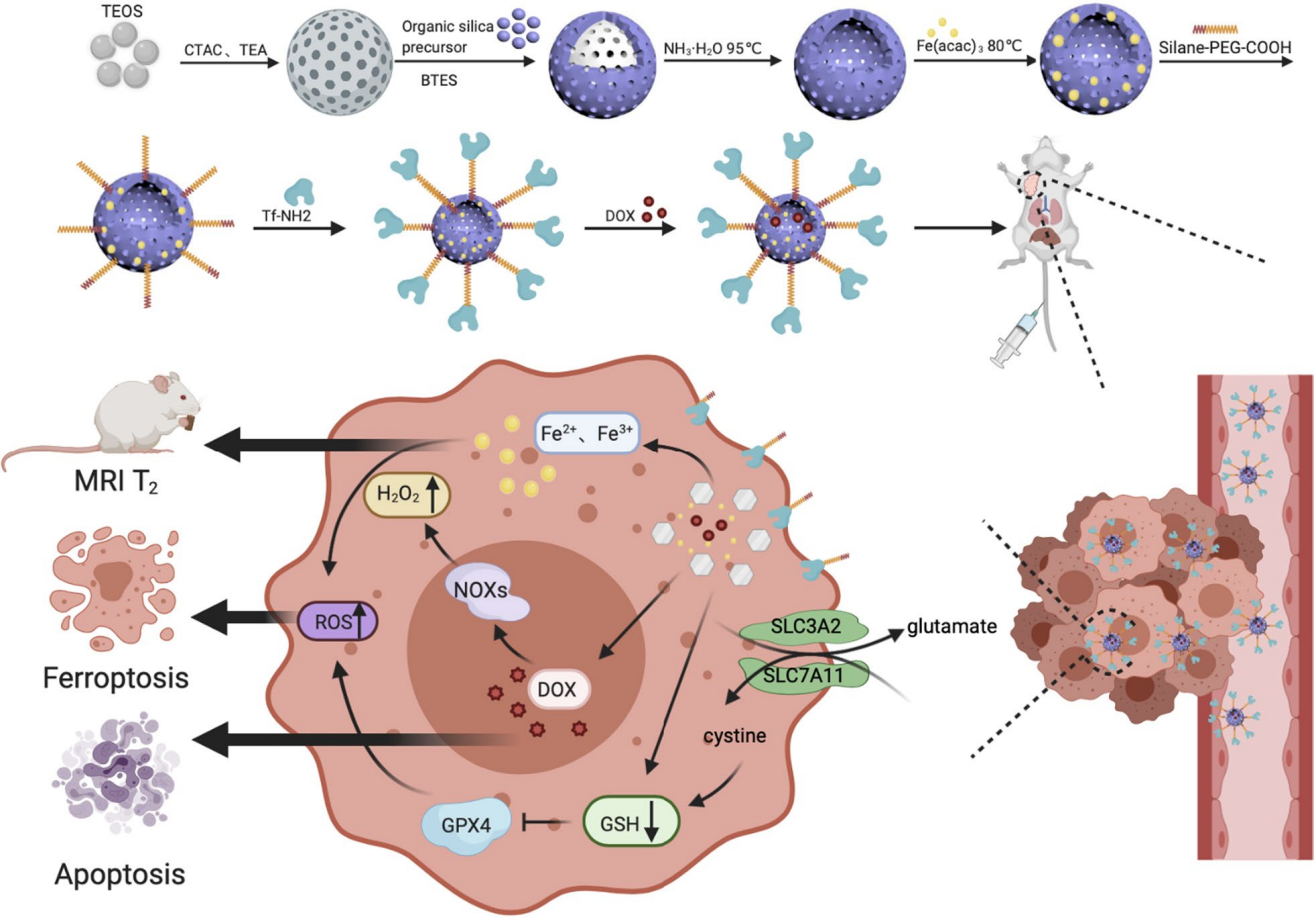

**Supplementary Information:**

The online version contains supplementary material available at 10.1186/s12951-021-01102-0.

## Background

Hepatocellular carcinoma (HCC) is the fourth leading cause of cancer-related mortality worldwide and the second leading cause of cancer-related deaths among men [1]. Early detection and monitoring programs are recommended. Nevertheless, only 30–40% of all patients are diagnosed at early stages and are eligible for curative treatments such as surgical resection, ablation, or transplantation [2]. Most untreated HCC cases progress to the intermediate or advanced stages and the median survival time is < 2y [3]. Doxorubicin (DOX) remains the most commonly administered chemotherapeutic agent against intermediate or advanced HCC [4]. However, conventional chemotherapy is ineffective against HCC, as this type of tumour is insensitive to chemotherapeutic agents [5, 6]. Hence, novel treatment approaches are required to supplement existing therapeutic options and improve treatment efficacy.

Ferroptosis is a recently discovered form of programmed cell death. It relies upon iron and reactive oxygen species (ROS) to induce lipid peroxidation [7]. Ferroptosis depends on chemically reactive and highly harmful hydroxyl radicals (•OH) to kill tumour cells. The •OH are generated by catalysing endogenous hydrogen peroxide (H_2_O_2_) via Fe^2+^/Fe^3+^ from the Fenton reaction [8, 9]. The unstable iron pool in cells catalyses ROS production through the Fenton reaction and is an effective type of chemodynamic therapy (CDT). Unlike photodynamic and sonodynamic therapy, CDT requires neither external energy nor oxygen. Hence, free radical-mediated tumour therapy has a far wider application range than other modalities [10, 11]. Cancer cells with therapy-resistant states are relatively more sensitive to ferroptosis than those without them [12]. Thus, therapy-resistant tumour cells are relatively more likely to be killed by ferroptosis than therapy-sensitive cancer cells [13]. Unfortunately, this approach has poor therapeutic efficacy as it is associated with relatively low iron concentrations, insufficient endogenous H_2_O_2_, and enhanced tumour antioxidant defence capability [14, 15].

H_2_O_2_ levels in tumour cells are not enough to produce the •OH levels required for satisfactory CDT efficacy [16, 17]. To increase its concentration in the tumour microenvironment (TME), H_2_O_2_ could be directly delivered to the tumour area via nanocarriers [18]. Another strategy is to generate H_2_O_2_ rapidly and efficiently by delivering glucose and oxygen to the tumour tissue [19, 20]. However, the former cannot continuously supply H_2_O_2_ whilst the latter is limited by the intrinsic hypoxia of the internal tumour tissue. These constraints lower the therapeutic efficacy of CDT in solid tumours. Moreover, high glutathione (GSH) concentrations are characteristic of TME. GSH is an important cellular antioxidant that can eliminate ROS and reduce CDT efficacy [21, 22]. Therefore, simultaneous H_2_O_2_ introduction and GSH elimination in a nanoplatform should result in an effective CDT.

DOX promotes tumour cell death by inhibiting DNA replication and generating H_2_O_2_ by activating nicotinamide adenine dinucleotide phosphate oxidases (NOXs) [23]. DOX treatment increases the levels of H_2_O_2_ which is the Fenton reaction substrate. Thus, DOX indirectly promotes ferroptosis. A combination of DOX and Fe^2+^/Fe^3+^ should, therefore, efficiently induce CDT. Fe^2+^/Fe^3+^ redox pair-based Fenton reaction-mediated biocatalytic tumour therapy has been widely used for tumour-specific iron delivery [24, 25]. Fe^2+^ also triggers ferroptosis by non-enzymatic lipid peroxidation and lipid auto-oxidation [26, 27]. Ferroptosis induced by Fe^2+^ can circulate in the membrane via the Haber–Weiss reaction. Thence, newly emerging lipid radicals attack adjacent polyunsaturated fatty acids and set off other lipid radical chain reactions [28]. This automatic amplification process is catalysed by Fe^2+^ and can extend membrane and ferroptotic damage after the failure of the molecular protection mechanism controlling lipid peroxidation. However, there are a few reports on the application of ferroptosis-based tumour treatment by regulation of ferroptosis-associated processes such as Fe^2+^ oxidation. Therefore, the development of a ferroptosis-based nanoplatform with tumour targeting and controllable pharmacokinetic properties could have important clinical significance in the enhancement of antitumour efficacy and the reduction of health risks [29].

Mesoporous silica nanoparticles (MSN NPs) have attracted increased attention as potential drug carriers for biomedical applications [30, 31]. Inorganic nanomaterials have higher physicochemical stability and versatility but lower biocompatibility and biodegradability than organic nanomaterials [32, 33]. Therefore, the construction of an organic/inorganic hybrid material nanoplatform should combine the advantages of both modalities whilst mitigating their respective shortcomings. Hence, this type of nanoplatform could have broad clinical applications [34]. Hollow biocompatible, biodegradable mesoporous organosilica nanoparticles (HMON NPs) have been developed via the selective introduction of bis[3-(triethoxysilyl)propyl]tetrasulphide (BTES) [35]. The GSH-responsive biodegradability and controlled release of antitumour drugs by thioether-hybridised HMON NPs could efficiently deliver DOX to target tumour cells and tissues.

Based on the properties of introduced Fe^2+^/Fe^3+^ and Fe‐coordination proteins, doping biocompatible Fe^2+^/Fe^3+^ into a silica framework (Fe-HMON NPs) could improve nanoplatform biodegradability [36]. However, traditional iron delivery nanomaterials may cause significant side effects when applied in vivo as they are mainly transported through enhanced permeability and retention effects [37]. Transferrin is an endogenous protein that ferries Fe^3+^ to cells overexpressing the transferrin receptor [38]. As HCC cells have abnormal iron metabolism, they overexpress transferrin receptors [39]. Grafting naturally occurring transferrin onto the surfaces of Fe-HMON NPs via polyethylene glycol (PEG) creates a target ligand (Fe-HMON-Tf NPs), thereby reducing phagocytosis in the reticuloendothelial system, retaining high solid tumour permeability and retention effects, and enhancing specific nanoplatform targeting [40]. Fe-based nanoparticles are also used as T2-weighted magnetic resonance imaging (MRI) contrast agents [41]. Fe-HMON-Tf NPs are superparamagnetic, and their T2-MRI performance facilitates therapeutic guidance and monitoring.

Here, we have proposed an intelligent iron-doped, hollow mesoporous organosilica nanoplatform that self-generates H_2_O_2_ and eliminates GSH for efficient ferroptosis-based CDT. In this manner, it inhibits tumour growth via sequential TME-activatable reactions. Iron-doped mesoporous silica nanoparticles have wide cavities that effectively load DOX. The latter is both a chemotherapeutic agent and a generator of H_2_O_2_ in tumour cells. Surface-modified transferrin is specific to the upregulated transferrin receptors in HCC cells and facilitates targeted tumour delivery and high intracellular DOX accumulation. The iron-doped hollow mesoporous organosilica nanoplatform can disintegrate under the action of abundant GSH in the TME, trigger rapid DOX release, enhance tumour inhibition efficacy, reduce toxic effects, and improve tolerance. In situ GSH consumption, the Fe^2+^/Fe^3+^ Fenton reaction, and H_2_O_2_ supply by DOX can trigger HCC cell ferroptosis. This mechanism reflects coordinated chemotherapy and CDT cascade. This multifunctional nanoplatform could reverse drug resistance in translational therapy, inhibit HCC recurrence and metastasis, and integrate accurate diagnosis, efficacious treatment, and real-time monitoring in clinical cancer medicine.

## Methods

### Materials

Cetyltrimethylammonium chloride (CTAC), triethanolamine (TEA), bis[3-(triethoxysilyl)propyl]tetrasulphide (BTES), tetraethyl orthosilicate (TEOS), ferrous acetylacetonate (Fe(acac)_2_) and transferrin were purchased from Sigma-Aldrich (MO, USA). Doxorubicin (DOX), deferiprone, urea, 3-(4,5Dimethylthiazol-yl)-2,5Dimethylthiazol-2-yl)-2,5diphenyltetrazolium bromide (MTT), *n*-(3-dimethylaminopropyl)-*n*′-ethylcarbodiimide hydrochloride (EDC), *n*-hydroxysuccinimide (NHS), L-Glutathione (GSH), silane–PEG-COOH (Mw = 2000) were purchased from Aladdin Reagent Database Inc (Shanghai, China). 4ʹ,6–diamidino-2-phenylindole (DAPI), indocyanine green (ICG) was obtained from Tokyo Chemical Industry (Tokyo, Japan). Foetal bovine serum (FBS), phosphate buffered saline (PBS), trypsin–EDTA and Dulbecco’s modified eagle’s medium (DMEM) were obtained from Gibco (USA). All other chemicals and solvents were of analytical or chromatographic grade.

### Synthesis of mesoporous organosilica nanoparticles (HMON NPs)

To start with, CTAC aqueous solution (20 g) and TEA aqueous solution (3.5 g) were first mixed and stirred at 80 °C for 15 min and TEOS (1 ml) was added dropwise for 1 h reaction. Subsequently, add a mixture of TEOS (0.5 ml) and BTES (1 ml) for another 4 h. Afterwards, the products were washed with ethanol several times and dispersed in methanol (30 mL) with NaCl (1wt.%) to extract the template. This step was repeated at least three times, each time at least 12 h to ensure the template was removed completely. The final HMON NPs were obtained by ammonia-assisted selective etching strategy reacting for 3 h at 95 °C with a certain amount of ammonia solution and washed with ethanol several times.

### Synthesis of Fe-doped hollow mesoporous organosilica nanoparticles (Fe-HMON NPs)

HMON NPs (25 mg) and Fe(acac)_2_ (200 mg) were dissolved completely in urea ethanol solution (25 ml) and homogenised for 5 min under ultrasound treatment. Then, the mixture was reacted for 12 h with stirring at 80 °C. The resultant Fe-HMON NPs were collected by centrifugation and washed with ethanol–deionised water solution several times.

### Synthesis of Fe-HMON-PEG-Tf NPs

Fe-HMON NPs (20 mg) was dissolved into ethanol (30 ml), followed by the addition of silane–PEG-COOH (30 mg) with stirring under 78 °C for 12 h. After the reaction, Fe-HMON-PEG NPs were obtained after centrifugation and washed with ethanol several times. Then EDC (12 mg) and NHS (15 mg) were added to Fe-HMON-PEG NPs (20 mg) suspended in 20 ml PBS to activate the -COOH groups to bind to transferrin. The mixture was carried out in an Erlenmeyer flask with stirring at 37 °C for 4 h. The products were centrifuged with PBS three times to remove excess EDC and NHS. Then added transferrin solution (200ul, 1 mg/ml) to the products and reacted for 12 h at 37 °C with stirring. Fe-HMON-PEG-Tf NPs were collected by centrifugation and washed with PBS three times.

### Characterisation

The particle size and size distribution were measured by Dynamic light scattering (litesizer500, Anton-Paar, Austria). The morphology of the MSN NPs, HMON NPs and Fe-HMON NPs was observed by transmission electron microscopy (JEM-1200EX, JEOL, Japan). X-ray diffraction (D/MAX-2550 PC, Rigaku Inc., Japan) pattern was using Cu Kα radiation with 2θ range of 10°-80°. The valence state of iron analysis was performed on the x-ray photoelectron spectrometer (ESCALAB 250Xi, Thermo Fisher Scientific, UK). Fourier transform infrared spectroscopy (VECTOR22, Bruker, Germany) of nanoparticles was performed in the range from 400 to 4000 cm^−1^. The distributions and proportions of silicon (Si), oxygen (O), iron (Fe), and sulphur (S) were performed using energy-dispersive spectroscopy elemental mapping (X-MAX^n^65 T, Oxford, UK). The nitrogen adsorption/desorption experiment was tested by using a Micromeritics Tristar II analyser (TriStar II, Micromeritics, USA). The surface areas and average pore size distributions were calculated by Brunauer–Emmett–Teller (BET) and Barrett–Joyner–Halenda (BJH) methods.

### Drug loading and release profiles

The encapsulation of DOX by HMON-Tf NPs, Fe-HMON-PEG NPs and Fe-HMON-Tf NPs was prepared by mixing the DOX (3 mg) with nanoparticles (10 mg) in PBS solution under dark conditions for 24 h. After that, the unloaded DOX was removed by centrifugation and the supernatants were reserved for the calculation of loading efficiency of drugs.

1 $${\text{Loading content }} = \left( {{\text{TD}} - {\text{FD}}} \right)/{\text{TN}} \times { 1}00\%$$

2 $${\text{Encapsulation efficiency }} = \, \left( {{\text{TD}} - {\text{ FD}}} \right)/{\text{TD}} \times { 1}00\%$$

where TD is the total weight of DOX fed, FD is the weight of nonencapsulated free DOX, and TN is the weight of nanoparticles.

To investigate the dissociation of DOX@ Fe-HMON-Tf NPs in response to pH and GSH trigger. A certain concentration of DOX@Fe-HMON-Tf NPs was dispersed into buffer solutions with different pH (7.4, 6.8, and 5.5) and different GSH concentrations (5 mM and 10 mM). At predetermined time points, undissolved nanoparticles were removed by centrifuging at 12000 rpm for 15 min and the concentration of DOX in the supernatant was detected using a fluorescence spectrophotometer. And the content of iron in the supernatant was measured by an inductively coupled plasma mass spectrometry instrument (ICP-MS, ICAPRQICPMS, Thermo Fisher, USA).

### In vitro and in vivo MRI

The Fe concentration of Fe-HMON-Tf NPs determined by ICP-MS. Various Fe concentrations (0, 0.036, 0.072, 0.288, 0.576, 1.152 mM) were dispersed in deionised water in 1 mL Eppendorf tubes and measured with a 3 T MRI scanner (Discovery MR 750, GE, USA) same time to obtain T2-weighted imagines. The relaxation coefficients r2 was obtained by fitting plots of the inverse relaxation times 1/T2 (s − 1) and Fe concentration (mM).

T2-weighted MRI was performed on a 3 T MRI scanner with a small-animal coil. The tumours-bearing mice need to be anaesthetised before and after the tail vein injection of nanoparticles. Then using a fast spin-echo sequence to scan with repetition time 3000 ms, time to echo 80 ms, the field of view 40 × 40 mm, matrix size 250 × 250 and slice thickness 2 mm.

### MTT assay on the cytotoxicity of various nanosamples

HepG2 cell line and LO2 cell line were obtained from the Chinese Academy of Sciences cell bank (Shanghai, China). The HepG2 cells were plated in 96-well plates at a density of 10^5^U per well and cultured at 37 °C with 5% CO_2_ overnight. The culture media were replaced with fresh ones containing PBS, DOX, HMON-Tf NPs, Fe-HMON-Tf NPs, DOX@HMON-Tf NPs, DOX@Fe-HMON-PEG NPs, DOX@Fe-HMON-Tf NPs and the cells were cultured for 24 h. The equivalent concentrations of DOX were maintained at 0.25, 0.5, 1, 1.5, 2, 2.5, 3, 3.5 μg/ml. Then 20ul MTT solution (5 mg/ml) was added into each well and incubated for 4 h. Afterwards, carefully removed the media and added 50µL DMSO to each well with low-speed oscillation for 15 min to dissolve the formazan crystals. The OD value was measured using a Microplate reader (Bio-Rad, Model 680, USA) at a wavelength of 570 nm. On a similar note, media containing only HMON-Tf NPs or Fe-HMON-Tf NPs at various concentrations (0, 5, 10, 20, 50ug/ml) were also incubated with inoculated HepG2 cells for 24 h before the MTT cytotoxicity assays.

### Assess cellular uptake and intracellular iron levels of different nanosamples

The HepG2 cells were plated into 24-well plates at a density of 10^5^U per well and cultured at 37 °C with 5% CO_2_ overnight. When the cell confluence reached around 70%, fresh media containing PBS, DOX, HMON-Tf NPs, Fe-HMON-Tf NPs, DOX@HMON-Tf NPs, DOX@Fe-HMON-PEG NPs and DOX@Fe-HMON-Tf NPs were used to replace the exhausted medium. The nanosamples concentrations were maintained at 20 μg/ml and the equivalent concentrations of DOX were maintained at 2ug/ml, the culture periods were set to 24 h. The cells were washed with PBS three times after the culture was completed. Then fixed with 4% paraformaldehyde solution for 0.5 h, stained with DAPI for 15 min. Finally, confocal laser scanning microscopy (CLSM; SP8 TCS, Leica, Germany) was used for observation and analysis.

For the determination of intracellular iron levels, repeated the above steps and separated the cells by trypsin without EDTA-Na, then added cell lysate to lyse the cells and sonicated the resultant solution to ensure the cells are completely broken down. The iron level was detected by ICP-MS as above.

### Evaluation of the level of H_2_O_2_ in tumour cells

HepG2 cells were seeded in two six-well plates at a density of 10^5^U per well and cultured under a normoxic environment and a hypoxic environment (AnaeroPack, MGC, Japan) for 2d, respectively. The cells were then lysed with Laemmli Sample Buffer (Bio-Rad Laboratories, Hercules, CA, USA), and the total protein was quantified by electrophoresis with a bicinchoninic acid (BCA) protein kit (Beyotime Biotechnology, Shanghai, China) and 12% sodium dodecyl sulfate–polyacrylamide gel electrophoresis (SDS-PAGE). The proteins were then transferred from the gel to a polyvinylidene difluoride (PVDF) membrane (Immobilon P; EMD Millipore, Billerica, MA, USA) and blocked with primary and secondary antibodies. The images were captured on a molecular imager (ChemiDoc Touch Imaging System, Bio-Rad, USA). HIF-1α expression was analyzed by western blot to confirm that the cells were hypoxic. Using the same protocol as above to plated hypoxic HepG2 cells into 24-well plates. When the cell confluence reached around 70%, the media was replaced with fresh ones containing different concentrations of DOX (0.0625, 0.125, 0.25, 0.5, 1, 2, 5 and 10 μg/ml) in each well and cultured for 24 h. Then the fluorescent probe was added to each well except the blank control and further cultured for 20 min at 37 °C. The intracellular H_2_O_2_ level was examined using the standard Fluorimetric Hydrogen Peroxide Assay Kit (Sigma-Aldrich, USA) that the red fluorescent product had an excitation wavelength of 640 nm and an emission wavelength of 680 nm, which could be used for the observation and analysis by CLSM.

In addition, mice with 60mm^3^ tumours were divided into four groups (each with six mice) and injected intravenously with different concentrations of DOX (0, 3, 5 and 10 mg/kg). The mice were euthanized at 24 h or 48 h. Then the tumours were excised and frozen in liquid nitrogen. The H_2_O_2_ content in the tumour tissues was detected with a Fluorimetric Hydrogen Peroxide Assay Kit (Sigma-Aldrich) according to the manufacturer’s protocol.

### Evaluation of the intracellular lipid peroxides

HepG2 cells were plated into 12-well plates as above. When the cell confluence reached 70%, the media was replaced with fresh ones containing PBS, DOX, HMON-Tf NPs, Fe-HMON-Tf NPs, DOX@HMON-Tf NPs, DOX@Fe-HMON-PEG NPs, DOX@Fe-HMON-Tf NPs and cultured for 24 h. The concentration of nanosamples was 20 μg/ml and the equivalent DOX concentration was kept at 2 μg/ml. The intracellular level of lipid peroxides was monitored by culturing with Fluorometric Intracellular ROS Kit (Sigma-Aldrich) for 30 min and measured by the flow cytometry system (CytoFLEX, Beckman Coulter, USA). The CLSM observation also used the same experimental protocol. The foregoing experimental setup was also used to determine the malondialdehyde (MDA) content and measurements were taken using a Lipid Peroxidation Assay Kit (Sigma-Aldrich).

### Evaluation of the mitochondrial membrane potential

HepG2 cells were plated into 24-well plates and dealt with PBS, DOX, HMON-Tf NPs, Fe-HMON-T NPs, DOX@HMON-Tf NPs, DOX@Fe-HMON-PEG NPs, DOX@Fe-HMON-Tf NPs when the cell confluence reached 70%. The concentration of nanosamples and DOX were the same as above and cultured for 24 h. After the incubation, the mitochondria were stained with JC-1 dye and observed by CLSM. When the mitochondrial membrane potential was high, JC-1 dye aggregated in the matrix to form polymers, which can produce red fluorescence (Ex/Em = 585/590 nm); when the mitochondrial membrane potential was low, JC-1 dye existed as monomers and produced green fluorescence (Ex/Em = 510/527 nm).

### Analysis of the cell apoptosis

HepG2 cells were plated into 12-well plates at the density of 10^5^U per well. When the cell confluence reached 70%, the cells were dealt with fresh media containing PBS, DOX, HMON-Tf NPs, Fe-HMON-Tf NPs, DOX@HMON-Tf NPs, DOX@Fe-HMON-PEG NPs, DOX@Fe-HMON-Tf NPs and the incubation lasted for 24 h. The concentration of nanosamples and DOX were the same as above. The Annexin V-FITC/PI apoptosis detection kit (Sigma-Aldrich, USA) was used as the protocol to study cell apoptosis by flow cytometry.

### Determination of intracellular GSH and GPX-4 activity

HepG2 cells were plated in 12-well plates at the density of 10^5^U per well and cultured at 37 °C with 5% CO_2_ overnight. The cells dealt with fresh media containing PBS, DOX, HMON-Tf NPs, Fe-HMON-Tf NPs, DOX@HMON-Tf NPs, DOX@Fe-HMON-PEG NPs, DOX@Fe-HMON-Tf NPs and the incubation continued for 24 h. The concentration of nanosamples and DOX were the same as above. Then cell lysates were collected and measured according to the instructions of GSH and GSSG Assay Kit. The UV–vis spectrophotometer (TU-1800PC, Beijing Purkinje General Instrument Co., Ltd., China) was used to measure the absorbance at 412 nm to determine the GSH level.

For the determination of intracellular GPX-4 activity, repeated the above steps and collected the cell lysates. According to the manufacturer’s instructions, the M5 full-band multi-function microplate reader (SynergyMx M5, Molecular Devices, USA) was used to measure the absorbance at 340 nm.

### Western blotting analysis

HepG2 cells were plated into six-well plates at a density of 10^5^U per well and cultured until the cell confluence reached around 70%. Afterwards, the cells dealt with fresh media containing PBS, DOX, HMON-Tf NPs, Fe-HMON-Tf NPs, DOX@HMON-Tf NPs, DOX@Fe-HMON-PEG NPs, DOX@Fe-HMON-Tf NPs and the culture continued for 24 h. The concentration of nanosamples and DOX were the same as above. To determine the expression levels of Caspase-3 apoptotic proteins, DOX-activated related proteins NOX4 and GPX4 ferroptosis-related proteins. Then the cells were lysed, and the total protein was quantified by electrophoresis using the BCA protein kit (Beyotime, China) and 12% SDS–polyacrylamide gel electrophoresis. The protein was then transferred from the gel to a polyvinylidene fluoride membrane (Immobilon P, Millipore, USA) and blocked with primary and secondary antibodies. The image was captured on a molecular imager (ChemiDoc Touch Imaging System, Bio-Rad, USA).

### Fluorescence imaging for tracking nanosamples in vivo

The tumour model establishment plan was as follows. The model mice were divided into two groups (6 mice/group). Fe-HMON-PEG NPs and Fe-HMON-Tf NPs were labelled with ICG, and the equivalent ICG concentration of the nanosamples injected into the tail vein was maintained at 1 mg/kg. The mice were anaesthetised at different time points, and the in vivo distribution was observed through the IVIS spectral imaging system (Caliper, PerkinElmer, USA). Furthermore, the mice were euthanised after 24 h, and the major organs were collected and observed the fluorescence signals, including tumour, heart, liver, spleen, lung, and kidney.

### Tumour treatment and histology analysis

All animal experiments were performed following the care and use guidelines of the National Institutes of Health (NIH, USA) and approved by the Animal Experiment Committee of Zhejiang University. Balb/c nude mice (4–5 weeks) were purchased from Shanghai Silaike Laboratory Animal Co., Ltd. The HepG2 tumour models were established by injecting 100 μl of PBS containing 10^7^U of HepG2 cells into the subcutaneous tissue of the mice. When the tumour size reached 60mm^3^, 42 HepG2 tumour-bearing mice were randomly divided into seven groups (6 mice/group). The tumour volume was calculated as V = LW^2^/2 (L, the maximum diameter of the tumour; W, the minimum diameter of the tumour). Then, all samples were injected through the tail vein at an equivalent DOX concentration of 5 mg/kg, including PBS, DOX, HMON-Tf NPs, Fe-HMON-Tf NPs, DOX@HMON-Tf NPs, DOX@Fe-HMON-PEG NPs and DOX@Fe-HMON-Tf NPs. The injection was repeated every two days for several cycles, and the body weight and tumour volume of nude mice were recorded. After 21 days, all mice were euthanised and their white blood cells (WBC), red blood cells (RBC), hemoglobin (HGB), platelet (PLT) were counted and compared (Mindray, BC-2800Vet, China). And the levels of glutamic-pyruvic transaminase (ALT), glutamic-oxalacetic transaminase (AST), alkaline phosphatase (ALP), creatinine (CREA) and blood urea nitrogen (BUN) were assayed (Rayto, Chemray 240, China) to evaluate the biological safety of the nanosamples. The tumours and major organs were collected and fixed with 10% formalin for 24 h. The paraffin-embedded sections were stained with haematoxylin and eosin (H&E) to monitor the cytotoxicity induced by various nanosamples and tumour sections performed terminal deoxynucleotidyl transferase–mediated deoxyuridine triphosphate nick end labelling (TUNEL) staining to determine the treatment effect. For the survival analysis, 42 HepG2 tumour–bearing mice were processed using the above procedures. No injections were given after 21 days and the number of live mice in each group was recorded until day 60.

### Statistical analysis

SPSS ver. 26.0 (IBM Inc., USA) and GraphPad Prism ver. 9.0 (GraphPad Software, USA) were used to process all data. Quantitative experimental data are recorded as means ± SD. We used the two-tailed t-test or the Mann–Whitney U test to compare two groups and the Kruskal–Wallis test to perform multiple comparisons. Statistical significance was set at *P* < 0.05.

## Results and discussion

### TME-activatable iron-doped nanoplatform construction and characterisation

**Fig. 1:**
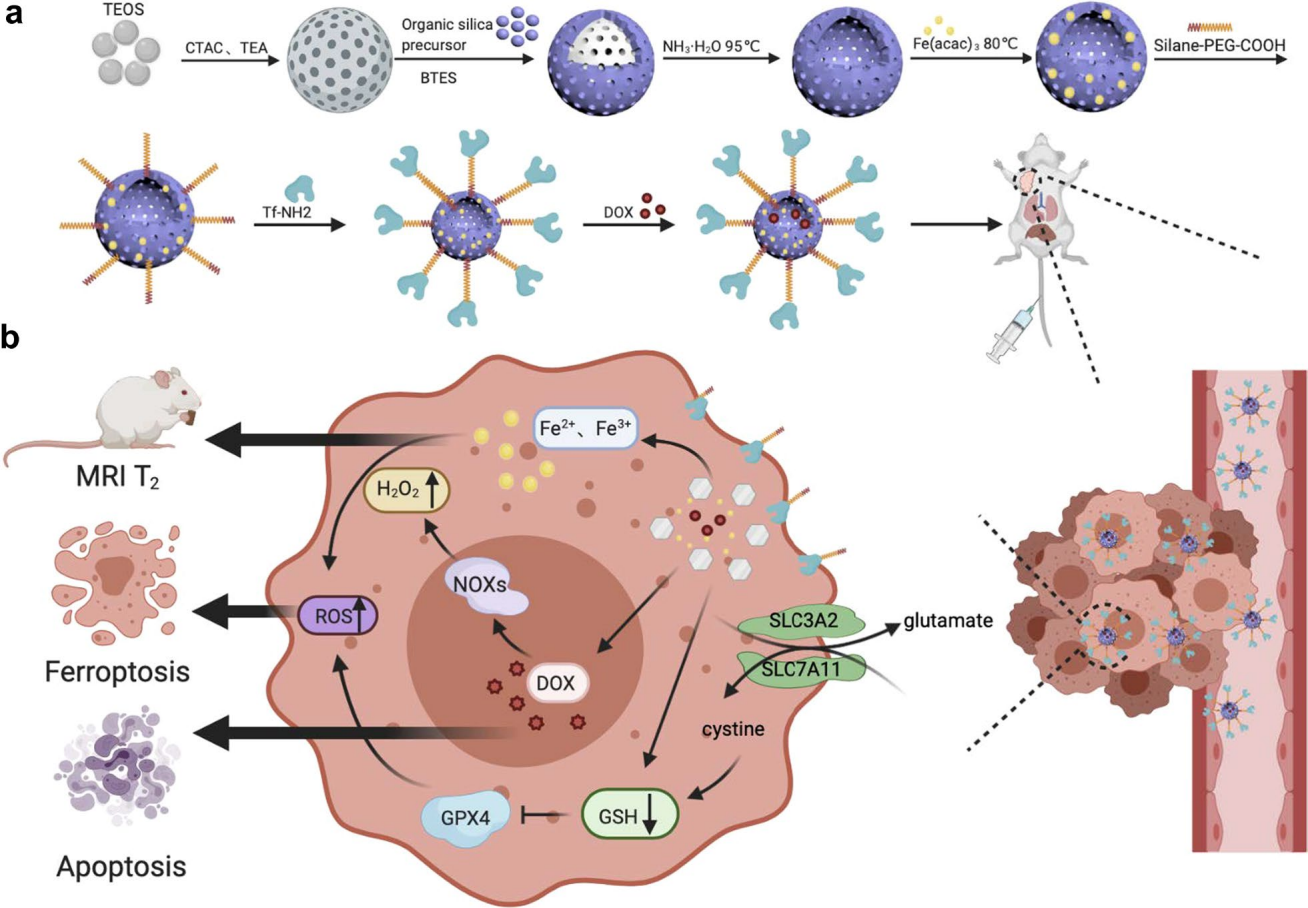
Synthesis and biomedical application of DOX@Fe-HMON-Tf NPs. **a** Schematic illustration of preparation of DOX@Fe-HMON-Tf NPs. **b** Schematic diagram of treatment and monitoring process of DOX@Fe-HMON-Tf NPs

The synthetic scheme and biomedical application of the tumour-targeting, TME-activatable, iron-doped, hollow mesoporous organosilica nanoplatform are illustrated in Fig. 1a and b. In brief, the construction of thioether hybrid mesoporous silica nanoparticles with a core/shell structure (HMON NPs) was based on the principle of chemical homology through BTES and tetraethoxysilane co-hydrolysis and co-condensation. CTAC and TEA were the structure‐directing agent and alkaline catalyst, respectively [35, 42]. The Si–C bond within the shell was more stable and stronger than the Si–O bond in the core. Hence, mild ammonia was used as the etching agent instead of strong acid/alkali (HF/NaOH) to obtain disulphide-bridged HMON NPs [43]. During the hydrothermal treatment, Fe(acac)_2_ reacted with the silicon-containing oligomers (dissolution process) that were released to form a Fe-doped silica layer on the HMON NP surface (growth process). This process was called "dissolution-growth" and entailed the strategy used to prepare Fe-HMON NPs.

**Fig. 2:**
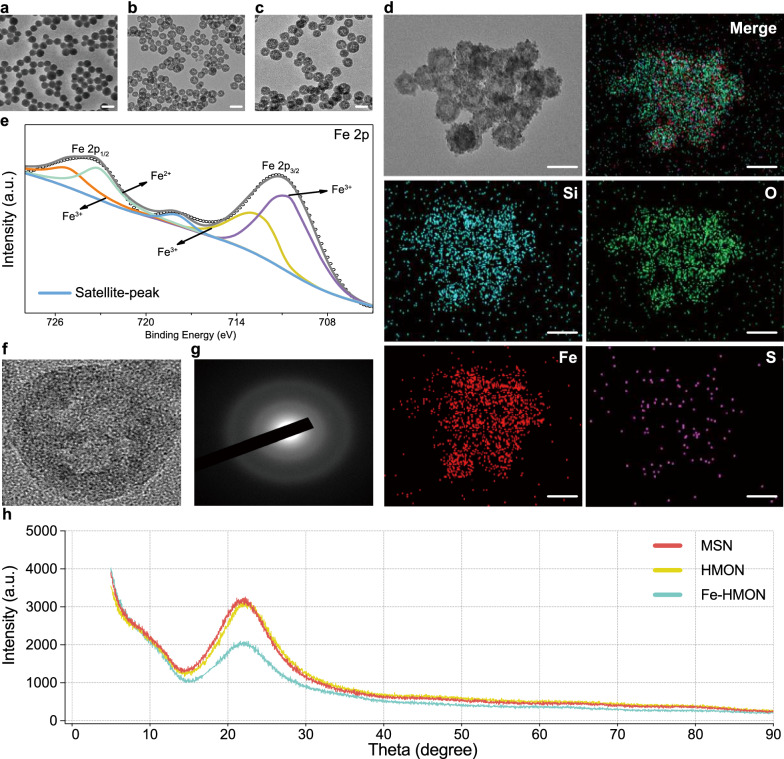
Structural and compositional nanoplatform characterisations. **a–c** TEM images of unetched HMON NPs, HMON NPs, and Fe-HMON NPs. Scale bar = 100 nm. **d** High-resolution TEM images of Fe-HMON NPs and elemental mapping (Si, O, Fe, and S) in Fe-HMON NPs. Scale bar = 50 nm. **e** Fe2p X-ray photoelectron spectroscopy (XPS) spectra of Fe-HMON NPs. **f** High-resolution TEM images of Fe-HMON NPs. **g** Selected-area electron diffraction patterns of Fe-HMON NPs. **h** XRD patterns of MSN NPs, HMON NPs, and Fe-HMON NPs

Transferrin was grafted by silane-PEG-COOH onto the surfaces of the Fe-HMON NPs to enhance targeting and generate Fe-HMON-Tf NPs. DOX was loaded into the Fe-HMON-Tf NPs through charge adsorption and complexation with Fe^2+^/Fe^3+^. Loading capacity up to 20.21% and complexation can reduce sensitivity to oxidative stress before Fe^2+^ is released into the cell. The transmission electron microscopy (TEM) images showed that all unetched HMON NPs, HMON NPs, and Fe-HMON NPs were spherical and have an average size of ~ 40 nm (Fig. 2a–c). Nanoparticle size did not significantly change after Fe doping. Dynamic light scattering (DLS) showed that the average hydrodynamic diameters of the Fe-HMON-PEG NPs and Fe-HMON-Tf NPs were ~ 56 nm and ~ 71 nm, respectively (Additional file 1: Fig. S1a). The N_2_ adsorption–desorption isotherms disclosed the presence of mesopores within the HMON NPs and Fe‐HMSN NPs. The Brunauer–Emmett–Teller (BET) surface area of the HMON NPs was 352.2 m^2^ g^−1^ and the pore size was ~ 4.1 nm. The BET surface area of the Fe-HMON NPs was 292.2 m^2^ g^−1^ and the pore size was ~ 3.3 nm (Additional file 1: Fig. S1c and d). Fe-HMON NPs had relatively large surface areas and mesopore sizes allowing for sufficient encapsulation of various drug loads. Fourier transform infrared (FTIR) spectroscopy confirmed the changes in chemical composition after each modification step (Additional file 1: Fig. S2b). The strong, broad peaks at 1,120 cm^−1^ were attributed to the Si–O bond whilst the peak at 485 cm^−1^ confirmed the presence of disulphide bonds in the nanostructure. The stretching vibration of the carboxyl C = O bond that formed after grafting the PEG created a new peak at 1,660 cm^−1^. The amide I and amide II bands at 1,562 cm^−1^ and 1,720 cm^−1^, respectively, suggested the successful introduction of protein molecules in the transferrin-coated nanoparticles. UV–vis spectroscopy** (**Additional file 1: Fig. S2a) and zeta potential analysis (Additional file 1: Fig. S1b) confirmed the changes caused by modification of the nanoplatform surface properties.

We also used energy-dispersive spectroscopy (EDS) to study the elemental composition of the Fe-HMON NPs. We identified the distributions of Si, O, Fe, and S in the nanostructure (Fig. 2d) and demonstrated the successful introduction of iron in the silica framework. Inductively coupled plasma mass spectrometry (ICP-MS) indicated that the amount of engineered iron was 27.30%. To predict the stabilisation efficiency of the Fe^2+^ in Fe-HMON NPs on potential oxidative stress in vivo, we quantified the iron valence distribution by comparing the fitting peak area via X-ray photoelectron spectroscopy (XPS). The fitting results of the Fe 2p3/2 spectra showed two characteristic peaks. The peaks at 709.5 eV and 712.9 eV were attributed to Fe^2+^ and Fe^3+^, respectively (Fig. 2e). Hence, Fe^2+^ predominated in Fe-HMON NPs as its fitting peak area was much higher than that of Fe^3+^. The Fe^2+^ in the Fe-HMON NPs framework had high oxidation resistance because of chemical bonding and was highly beneficial for inducing ferroptosis in vivo. High-resolution TEM and selected area electron diffraction characterisations (Fig. 2f and g) showed that the Fe-HMON NPs were amorphous. And the x-ray diffraction (XRD) pattern of the Fe-HMON NPs disclosed a typical amorphous phase and a visible broad peak at 2θ = 23° (Fig. 2h) compared with MSN NPs and HMON NPs.

### TME-activatable iron-doped nanoplatform release behaviour

**Fig. 3:**
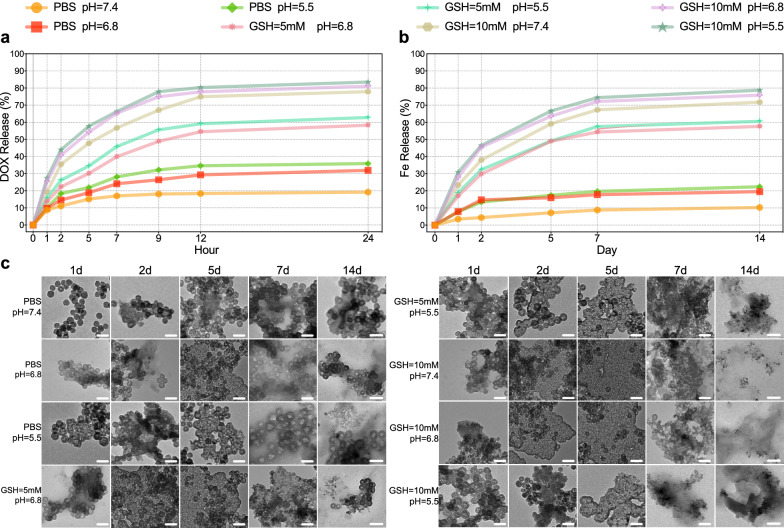
Simulated TME triggers release characteristics of DOX and iron ions, and appearance of nanoplatforms under TEM. **a** The DOX release profiles for DOX@Fe-HMON-Tf NPs with or without various GSH concentrations at pH 7.4, 6.8 and 5.5. **b** Accumulated degradation profiles of Fe species in PBS at different pH and various GSH concentrations. **c** TEM images of DOX@Fe-HMON-Tf NPs in PBS with or without various GSH concentrations, at neutral (pH 7.4) and acidic (pH 6.8 and 5.5) conditions at different time intervals (1d, 2d, 5d, 7d, and 14d). Scale bar = 100 nm

Thioether hybrid HMON NPs were selected for biomedical applications as they have reductive responsive biodegradability because of GSH-triggered, in-frame disulphide bond cleavage [35]. Iron and protein coordination can enrich the silica framework with reaction sites and accelerate silica framework biodegradation [44]. The effective use of the prepared composite nanoplatform to achieve ferroptosis-based tumour therapy depends on the release of the DOX@Fe-HMON-Tf NP contents. Considering more acidic pH in endosomes (pH 5.0 − 6.5) and lysosomes (pH 4.5 − 5.0) of tumour cells than the physiological environment (pH 7.4) [45]. We investigated the release characteristics of DOX and Fe^2+^/Fe^3+^ by incubating DOX@Fe-HMON-Tf NPs in buffer solution. DOX@Fe-HMON-Tf NPs exhibited excellent chemical stability at pH 7.4 and the total DOX leakage was < 20% after 24 h incubation. When the nanosamples were cultured at pH 6.8 and 5.5 for 24 h, however, the release percentage was ~ 30%. Therefore, the nanoplatform was slightly less sensitive to acidic environments (Fig. 3a). The release percentage was ~ 65% after the nanosamples were incubated in 5 mM GSH at pH 6.8 and 5.5 for 24 h. The release percentage was ~ 80% after the nanosamples were incubated in 10 mM GSH solution at all three pH for 24 h. The disulphide bonds in the Fe-HMON-Tf NPs framework tended to be cleaved in reducing TME. Fe-HMON-Tf NPs gradually degraded in a mimic GSH solution [46]. ICP-MS demonstrated similar but slower iron release rate trends. After 7d, the iron release percentage was < 20% at all three pH. It increased to ~ 60% in 5 mM GSH at pH 6.8 and 5.5 and ~ 75% in 10 mM GSH at all three pH (Fig. 3b). TEM of the incubated nanoparticles displayed that the severity of nanoplatform structure collapse was positively correlated with the cumulative iron release. When the culture period in 5 and 10 mM GSH was extended to 7d, the nanoplatform fragmented. However, the fragments gradually disappeared when the culture was prolonged to 14d (Fig. 3c). The TEM images validated the degradability of the composite nanoplatform under physiological conditions. We also found that DOX@Fe-HMON-Tf NPs exhibited time-dependent biodegradability in foetal bovine serum (FBS). Numerous nanoparticle degradation products were generated after 5d because of the coordination effects of the protein on Fe^2+^/Fe^3+^ in FBS (Additional file 1: Fig. S3). Biodegradation of the silica framework was accelerated as iron was extracted from the DOX@Fe-HMON-Tf NPs [44].

### In vitro evaluation of the nanosample cytotoxicity profiles

LO2 cells and HepG2 tumour cells were co-incubated with various nanosamples for 24 h to validate nanosamples biocompatibility and therapeutic efficacy. HMON-Tf NPs showed no substantial cytotoxicity towards LO2 cells whereas Fe-HMON-Tf NPs displayed concentration-dependent cytotoxicity towards LO2 cells (Additional file 1: Fig. S4a). In the presence of 50 μg/mL Fe-HMON-Tf NPs, HepG2 cell viability was reduced to 37.6% compared with the control. By contrast, HMON-Tf NPs were not cytotoxic even at high concentrations (Additional file 1: Fig. S4b). The Fenton reaction triggered by Fe^2+^/Fe^3+^ might have generated highly toxic free radicals that were harmful to the HepG2 cells.

3 $${\text{Fe}}^{{{3} + }} + {\text{H}}_{{2}} {\text{O}}_{{2}} \to {\text{Fe}}^{{{2} + }} + {\text{HO}}_{{2}} \cdot + {\text{H}}^{ + }$$

4 $${\text{Fe}}^{{{2} + }} + {\text{H}}_{{2}} {\text{O}}_{{2}} \to {\text{Fe}}^{{{3} + }} + \cdot {\text{OH}} + {\text{HO}}^{ - }$$

5 $${\text{Fe}}^{{{2} + }} + {\text{HO}}_{{2}} \cdot \to {\text{Fe}}^{{{3} + }} + {\text{HO}}_{{2}}^{ - }$$

6 $${\text{Fe}}^{{{3} + }} + {\text{HO}}_{{2}} \cdot \to {\text{Fe}}^{{{2} + }} + {\text{O}}_{{2}} + {\text{H}}^{ + }$$

Abundant Fe^3+^ in the nanoplatform could be reduced to Fe^2+^ when intracellular GSH is upregulated. This reaction enhances ROS production and CDT effects [47].

### Analysis of nanosample MRI capability

**Fig. 4:**
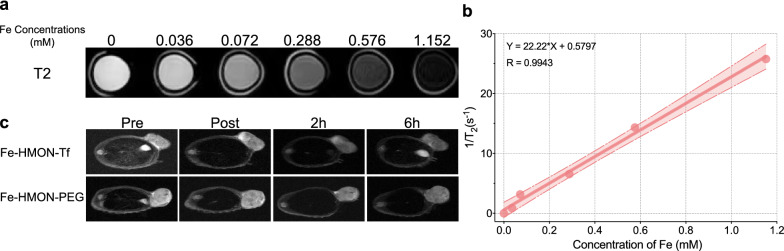
Nanoplatform T2 MRI capability. **a** T2-weighted solution MRI images of DOX@Fe-HMON-Tf NPs at different Fe concentrations (0, 0.036, 0.072, 0.072, 0.288, 0.576, and 1.152 mM). **b** T2 relaxation vs. Fe concentration of DOX@Fe-HMON-Tf NPs in PBS. **c** In vivo T2-weighted MRI of HepG2 tumour-bearing nude mice before and after intravenous DOX@Fe-HMON-Tf NPs administration for prolonged time intervals

The superparamagnetic framework endued DOX@Fe-HMON-Tf NPs with potential T2-weighted MRI contrast characteristics that favour tumour treatment guidance and monitoring. The in vitro MRI results showed that the nanoplatform had r^2^ relaxivity = 22.2 mM^−1^ s^−1^ (Fig. 4a and b). HepG2 tumour-bearing nude mice were assayed before intravenous DOX@Fe-HMON-Tf NP administration, at the time of injection (0 h), and at 2 h and 6 h post-treatment. After the DOX@Fe-HMON-Tf NP injection, the tumour tissue signal was significantly reduced possibly because of the targeting ability and T2-weighted MRI performance of the nanosamples (Fig. 4c). This excellent T2-weighted MRI performance of the Fe-HMON-Tf nanoplatform suggested that it could be promising in therapeutic guidance and monitoring. Theoretically, it is possible to perform T1-weighted MRI because the iron in the nanoplatform is converted into iron ions in the TME. However, the actual situation was that the T1-weighted MRI performance was not good. This may be due to the insufficient time required for the degradation of nanoparticles to iron ions to meet imaging standards.

### Evaluation of nanosamples uptake efficiency in HepG2 cells

**Fig. 5:**
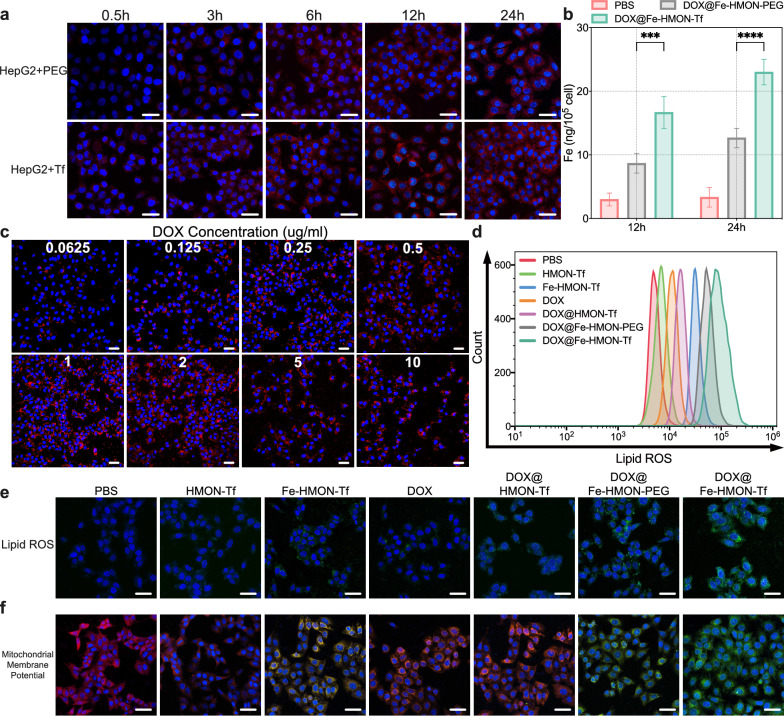
In vitro uptake and ROS generation capacity of nanosamples. **a** CLSM images of HepG2 cells incubated with DOX@Fe-HMON-PEG NPs and DOX@Fe-HMON-Tf NPs for 24 h. Blue and red indicate nuclei and DOX-loaded nanoparticles, respectively. **b** ICP results for intracellular iron levels in HepG2 cells incubated with DOX@Fe-HMON-PEG NPs or DOX@Fe-HMON-Tf NPs for 24 h. ***P < 0.001; ****P < 0.0001, Kruskal–Wallis test; mean ± SD. **c** CLSM images of intracellular H_2_O_2_ levels in HepG2 cells incubated with various DOX concentrations for 24 h. **d**–**e** Flow cytometry and CLSM of intracellular lipoperoxide levels in HepG2 cells incubated with PBS, DOX, HMON-Tf NPs, Fe-HMON-Tf NPs, DOX@HMON-Tf NPs, DOX@Fe-HMON-PEG NPs or DOX@Fe-HMON-Tf NPs for 24 h. **f** CLSM of changes in mitochondrial membrane potential of HepG2 cells incubated with PBS, DOX, HMON-Tf NPs, Fe-HMON-Tf NPs, DOX@HMON-Tf NPs, DOX@Fe-HMON-PEG NPs, or DOX@Fe-HMON-Tf NPs for 24 h. Scale bar = 50 μm

The CLSM images revealed intracellular red fluorescence of DOX molecules in HepG2 cells which indicated that DOX@Fe-HMON-Tf NPs efficiently delivers DOX into these cells. DOX@Fe-HMON-PEG NPs without transferrin modification was partially absorbed by the HepG2 cells. Although HepG2 cells exhibited higher DOX accumulation in the presence of DOX@Fe-HMON-Tf NPs (Fig. 5a), the difference between the two was not prominent, which may be due to the function of PEG and the longer incubation time. We previously demonstrated that the transferrin receptor was overexpressed in HepG2 cells [48]. We also used ICP-MS to study nanosample-induced intracellular iron accumulation, which was more representative. Compared with the DOX@Fe-HMON-PEG group, the iron concentration in the DOX@Fe-HMON-Tf NP group was ~ 40% higher (Fig. 5b).

### The ability of DOX to induce H_2_O_2_ in vitro and in vivo

Ferroptosis is usually the direct result of lipid peroxide accumulation and depends on cellular iron overload. Simultaneous increases in the iron and H_2_O_2_ levels in tumour cells may effectively initiate and execute ferroptosis. Prior studies consistently showed that high intracellular H_2_O_2_ levels were attributed to intracellular NOX4 upregulation by DOX. [49]. Therefore, DOX-induced NOX4 activation might increase intracellular H_2_O_2_ production and diffuse lipid peroxidation. We first investigated the expression of HIF-1α analyzed by western blot to confirm the cells were hypoxic (Additional file 1: Fig. S5a). Then we assessed DOX-induced H_2_O_2_ production capacity by incubating hypoxic HepG2 cells with various DOX concentrations and monitoring intracellular H_2_O_2_ levels. Intracellular H_2_O_2_ significantly increased when the DOX concentration was 1 μg/mL to 2 μg/mL (Fig. 5c). However, when the concentration of DOX was too high, glucose-dependent NADPH synthesis was repressed [50] and NOX4 activation and H_2_O_2_ generation were reduced. The favorable in vitro efficacy of DOX at inducing H_2_O_2_ facilitated the subsequent investigation of its in vivo H_2_O_2_-inducing efficacy as well. In addition, hypoxia is one of the characteristics of the TME [51]. HepG2 tumour-bearing mice received different DOX concentrations and the effect was optimal at 5 mg/kg (Additional file 1: Fig. S5b). This dosage was used in the subsequent in vivo experiments.

### Nanosample-induced changes in intracellular lipid peroxides and mitochondrial membrane potential

The intracellular ROS levels did not significantly change after HepG2 cells were incubated with different concentrations of DOX (Additional file 1: Fig. S5c and d). By contrast, when HepG2 cells were co-incubated with iron-containing nanosamples, the intracellular ROS levels significantly increased. Flow cytometry revealed that ferroptosis was induced by DOX@Fe-HMON-Tf NPs and the absorption capacity of the Tf-modified nanoplatform was enhanced in tumour cells (Fig. 5d). Moreover, the DOX@Fe-HMON-Tf NPs were at the highest concentrations in the HepG2 cells (Fig. 5e). MDA is the terminal product of lipid peroxidation. DOX@Fe-HMOlN-Tf NPs exhibited the highest MDA levels of all groups (Additional file 1: Fig. S5e), which evidently supported the occurrence of lipid peroxidation. Elevated iron and MDA levels in tumour cells indicate a high probability that ferroptosis will occur [52].

Mitochondria are the energy-producing organelles of human cells. They also harbour multiple programmed cell death pathways. During ferroptosis, the mitochondrial membrane potential significantly changes in response to ROS accumulation. Here, we measured the mitochondrial membrane potential of HepG2 cells with a JC-1 detection kit. At elevated mitochondrial membrane potentials, JC-1 accumulates in the mitochondrial matrix and forms a polymer that can emit red fluorescence. At low mitochondrial membrane potentials, JC-1 is a monomer and emits green fluorescence. Hence, the severity of cell damage caused by various nanosample treatments can be assessed according to the colour changes in response to mitochondrial JC-1 staining. CLSM showed that HepG2 cells presented with yellow-green fluorescence after 24 h culture with free DOX (Fig. 5f). However, cells treated with DOX@Fe-HMON-Tf NPs exhibited strong green fluorescence. Thus, the mitochondrial membrane potential was highly negative because of the introduction of iron cations. This phenomenon suggests that mitochondria are highly sensitive to lipid peroxidation after ferroptosis induction.

### Efficacy and mechanism of dual ferroptosis/apoptosis tumour treatment

**Fig. 6:**
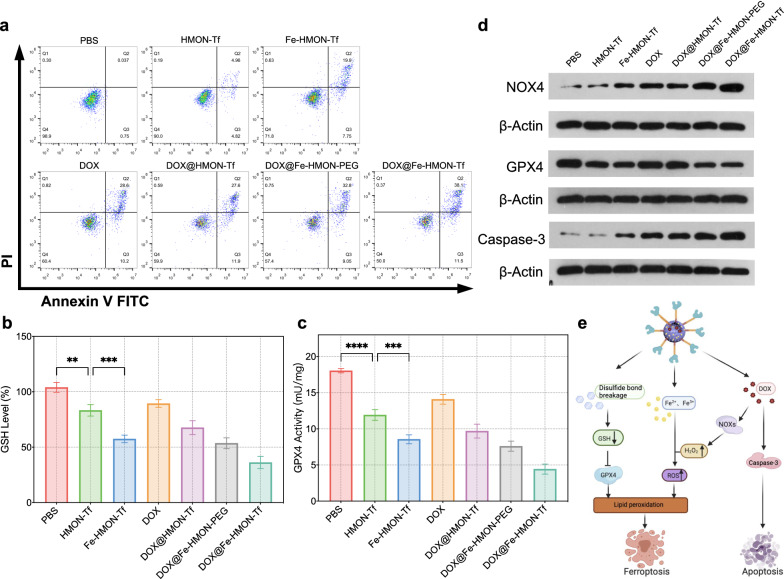
In vitro antitumour effect evaluation and mechanism exploration of the nanoplatform. **a** Flow cytometry of apoptosis levels in HepG2 cells incubated with PBS, DOX, HMON-Tf NPs, Fe-HMON-Tf NPs, DOX@HMON-Tf NPs, DOX@Fe-HMON-PEG NPs or DOX@Fe-HMON-Tf NPs for 24 h. **b** GSH levels in HepG2 cells incubated with PBS, DOX, HMON-Tf NPs, Fe-HMON-Tf NPs, DOX@HMON-Tf NPs, DOX@Fe-HMON-PEG NPs or DOX@Fe-HMON-Tf NPs for 24 h. **P < 0.01; ***P < 0.001, Kruskal–Wallis test; mean ± SD. **c** GPX4 activity in HepG2 cells incubated with PBS, DOX, HMON-Tf NPs, Fe-HMON-Tf NPs, DOX@HMON-Tf NPs, DOX@Fe-HMON-PEG NPs or DOX@Fe-HMON-Tf NPs for 24 h. ***P < 0.001; ****P < 0.0001, Kruskal–Wallis test; mean ± SD. **d** Western blot of key ferroptosis marker GPX4 expression and apoptosis markers NOX4 and Caspase-3 in HepG2 cells incubated with PBS, DOX, HMON-Tf NPs, Fe-HMON-Tf NPs, DOX@HMON-Tf NPs, DOX@Fe-HMON-PEG NPs or DOX@Fe-HMON-Tf NPs. **e** Proposed molecular mechanisms of nanoplatform-induced synergistic ferroptosis and apoptosis

The cytotoxicity of the composite nanoplatform to HepG2 cells was determined by MTT. In this assay, the equivalent DOX concentrations remained the same. Survival of the HepG2 cells in the DOX@Fe-HMON-Tf NPs group was as low as 43.1% whereas survival of the HepG2 cells in the free DOX group stayed as high as 55.3% after 24 h at a DOX concentration of 2 μg/mL (Additional file 1: Fig. S4b). Flow cytometry corroborated the findings of the MTT assay. Up to 38.1% of the HepG2 cells had died after 24 h culture with DOX@Fe-HMON-Tf NPs. By contrast, only 28.6% of the HepG2 cells had died after 24 h culture with free DOX (Fig. 6a). The foregoing results demonstrated that a combination of ferroptosis and DOX was superior to traditional chemotherapy regimens.

We then used various complementary technologies to elucidate the cell death mechanism associated with DOX@Fe-HMON-Tf NPs treatment. DOX treatment can upregulate the NOX4 and increase the H_2_O_2_ levels required for the in situ iron-mediated Fenton reaction [53]. In this manner, it promotes lipid peroxide accumulation and contributes to ferroptosis. Rupture of the disulphide bond in the nanostructure and the presence of ferric iron convert GSH to oxidised glutathione (GSSG). The order of GSH content was Fe-HMON-Tf NPs < HMON-Tf NPs < PBS. Therefore, the nanoplatforms entering the cells effectively consumed GSH (Fig. 6b). To ensure membrane integrity and minimise damage caused by ROS, GPX4 uses reduced GSH as a cofactor to convert lipid hydroperoxides into lipid alcohols. This reaction prevents iron-dependent, toxic lipid ROS formation and accumulation [54, 55]. Depleting the intracellular GSH pool to lower GPX4 activity and raise lipid peroxidation levels ultimately leads to ferroptosis. We investigated the changes in GPX4 expression and activity in HepG2 cells subjected to the nanosamples. Co-incubation with Fe-HMON-Tf NPs and HMON NPs bearing disulphide bond structures significantly reduced GPX4 activity (Fig. 6c) as well as protein expression levels (Fig. 6d) in HepG2 cells. Compared with Fe-HMON-Tf NPs, DOX@Fe-HMON-Tf NPs more strongly inhibited GPX4 activity and expression. Hence, DOX may be able to downregulate GPX4 and reduce Fe^2+^/Fe^3+^ and disulphide bonds, thereby permitting lipid peroxidation and ferroptosis. Moreover, caspase-3 upregulation was observed because simultaneous DOX delivery activated the caspase-mediated apoptosis pathway. These results indicated that the death of HepG2 cells cultured with DOX@Fe-HMON-Tf NPs was caused by a combination of ferroptosis and DOX-mediated apoptosis. DOX induced apoptosis and enhanced ROS in the nanoplatform. The molecular mechanism of synergistic ferroptosis and apoptosis induced by DOX@Fe-HMON-Tf NPs was also disclosed by western blotting (Fig. 6e).

### In vivo nanosample distribution patterns

**Fig. 7:**
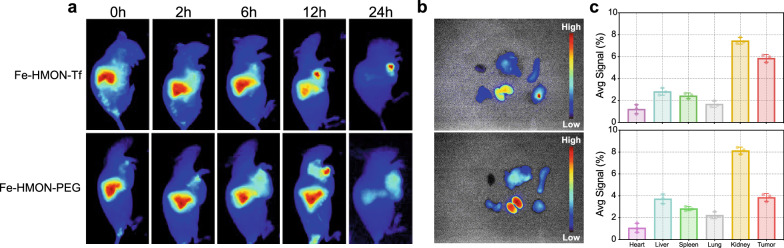
Fluorescence investigation of distribution patterns of nanoplatforms in tumour-bearing nude mice. **a** Fluorescence images of HepG2 tumour-bearing nude mice after intravenous injection with DOX@Fe-HMON-PEG NPs and DOX@Fe-HMON-Tf NPs at different time points. **b** Ex vivo fluorescence images of organs and tumours harvested from mice bearing HepG2 tumours at 24 h after treatment. **c** Fluorescence intensities of harvested tissues at 24 h post-injection. Mean ± SD

To determine nanosample tumour specificity, delivery efficiency, and in vivo distribution patterns after intravenous injection, the near-infrared-absorbing dye ICG was used to stain the nanosamples. Compared with DOX@Fe-HMON-PEG NPs, DOX@Fe-HMON-Tf NPs more effectively accumulated in the tumour tissues (Fig. 7a). Organs and tumours were harvested at 24 h after injection for fluorescence imaging and quantitative analysis. Total ICG fluorescence was more intense in the tumour tissues treated with DOX@Fe-HMON-Tf NPs than it was in the tumour tissues exposed to DOX@Fe-HMON-PEG NPs (Fig. 7b and c) possibly because surface transferrin modification occurred in response to the latter treatment. The fluorescence intensity of the kidneys in the two groups of organs was higher, which may imply the further study of the main metabolic pathway of the nanosamples in vivo.

### Evaluation of in vivo therapeutic efficacy of ferroptosis combined with chemotherapy

**Fig. 8:**
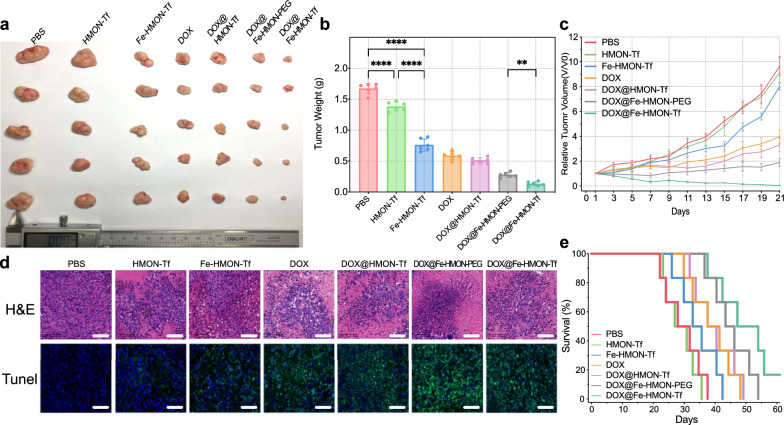
In vivo therapeutic nanoplatform efficacy. **a** Comparison of tumour tissues extracted from HepG2 tumour-bearing mice after 21d treatment with PBS, DOX, HMON-Tf NPs, Fe-HMON-Tf NPs, DOX@HMON-Tf NPs, DOX@Fe-HMON-PEG NPs or DOX@Fe-HMON-Tf NPs. **b** Final weights of tumour tissues extracted from HepG2 tumour-bearing mice after 21d treatment with PBS, DOX, HMON-Tf NPs, Fe-HMON-Tf NPs, DOX@HMON-Tf NPs, DOX@Fe-HMON-PEG NPs or DOX@Fe-HMON-Tf NPs. **P < 0.01; ****P < 0.0001, Kruskal–Wallis test. **c** Time-dependent changes in tumor volumes in HepG2 tumor-bearing mice. Mean ± SD. **d** Histological analysis of tumour slices stained with H&E and TUNEL after treatment with PBS, DOX, HMON-Tf NPs, Fe-HMON-Tf NPs, DOX@HMON-Tf NPs, DOX@Fe-HMON-PEG NPs or DOX@Fe-HMON-Tf NPs. Scale bar = 100 μm. **e** Survival rates of HepG2 tumour-bearing mice after 60d. n = 6

The antitumour ability of the nanosamples was evaluated using a mouse HepG2 tumour model. Six mice were assigned to each of the PBS, HMON-Tf NP, Fe-HMON-Tf NP, DOX, DOX@HMON-Tf NP, DOX@Fe-HMON-PEG, and DOX@HMON-Tf NP groups. The amounts of DOX and nanosamples and the treatment periods were the same for all groups. Based on the relative tumour sizes, tumour suppression was most evident in the DOX@HMON-Tf NPs group compared to the others (Fig. 8a). The HepG2 tumour weights were lowest (~ 0.13 g) after 21d DOX@Fe-HMON-Tf NPs treatment. By contrast, the relative tumour volume and weight were 4 and 0.58 g, respectively, in the free DOX group (Fig. 8b and c). Tumour suppression was stronger in the DOX@Fe-HMON-Tf NPs group than the DOX@Fe-HMON-PEG NPs group because the former had the superior active tumour-targeting ability. H&E and TUNEL staining showed that tumour cell death was most evident in the DOX @Fe-HMON-Tf NPs group (Fig. 8d). The foregoing data supported the hypothesis that the DOX@Fe-HMON-Tf NPs effectively inhibits tumour growth in animal models by combining ferroptosis and chemotherapy.

The observed weight changes in the mice during the experiment indicated potential side effects of the nanosamples (Additional file 1: Fig. S6a). The free DOX group presented with substantial weight loss at ~ 3d after administration and the final average weight was only 19.5 g. By comparison, the mean body weights of the DOX@Fe-HMON-PEG NPs and DOX@Fe-HMON-Tf NP groups remained at 20.3 g and 20.6 g, respectively, and resembled that of the PBS group. We also performed hematological and serum biochemical analysis. Relative to the PBS group, the DOX@Fe-HMON-Tf NPs group presented with similar WBC, RBC, HGB, and PLT counts (Additional file 1: Fig. S6b–e). Moreover, the ALT, AST, ALP, CREA and BUN levels were not comparatively higher in the DOX@Fe-HMON-Tf NP (Additional file 1: Fig. S6f–h). For this reason, nanotherapy was not expected to cause liver or kidney damage. Therefore, the nanoplatform had good biocompatibility and low therapeutic toxicity. Histological analysis and H&E staining of the major organs collected from each mouse group disclosed that free DOX severely damaged the myocardium. However, this toxic effect was not apparent in either the DOX@Fe-HMON-PEG NPs or the DOX@Fe-HMON-Tf NPs group (Additional file 1: Fig. S6i). However, some studies believed that ferroptosis plays a considerable role in DOX-induced cardiotoxicity[56]. Whether the tumour-bearing mice will have myocardial damage in the later stage under the intervention of this nanoplatform is worthy of attention. A survival analysis also underscored the superior efficacy and safety of the DOX@Fe-HMON-Tf NPs treatment (Fig. 8e). The median survival time of HepG2 tumour mice treated with DOX@Fe-HMON-Tf NPs was 50d which was much longer than those of the other groups. These findings suggest that the DOX@Fe-HMON-Tf NPs had good biocompatibility and therapeutic efficacy and might, therefore, be useful in clinical application.

## Conclusions

In the present study, we constructed a tumour-targeting theranostic nanoplatform by incorporating tetrasulphide bonds and the active sites of iron into a silica framework and loading DOX to generate H_2_O_2_ in situ. The nanoplatform protected Fe^2+^ from the oxidative regulation of the biological environment before it reached the specific tumour site. Hence, it optimised therapeutic antitumour efficacy. DOX@Fe-HMON-Tf NPs had surface-modified transferrin, and transferrin receptor-overexpressing liver cancer cells had a high affinity for them. In this manner, the nanoparticles could target and accumulate in the tumour tissues. The nanoplatform could be activated by the GSH-rich TME and induce tumour cell death by complementary ferroptosis and apoptosis mechanisms. The superparamagnetic framework of the nanoplatform could also be used in T2 modal MRI-guided hepatocarcinoma diagnosis. The nanoplatform designed and tested here may overcome the limitations of traditional antitumour regimens and have magnetic resonance imaging capability for diagnostic and therapeutic efficacy monitoring. Hence, this novel approach can integrate the precise diagnosis, efficacious treatment, and real-time monitoring of hepatocellular carcinoma in clinical medicine.

## Supplementary Information

**Additional file 1: Fig. S1**. Nanoplatform characterisation. (a) Sizes and (b) ζ-potentials of HMON NPs, Fe-HMON NPs, Fe-HMON-PEG NPs, and Fe-HMON-Tf NPs. (c) Nitrogen sorption isotherms and (d) pore size distribution curves for Fe-HMON NPs. **Fig. S2**. Nanoplatform composition. (a) Coomassie Blue analysis of particles after transferrin modification with UV–vis spectroscopy. Fe-HMON-Tf NPs stained blue while Fe-HMON-PEG NPs stained green. (b) FTIR spectra of HMON NPs, Fe-HMON NPs, Fe-HMON-PEG NPs, and Fe-HMON-Tf NPs. **Fig. S3**. TEM images of DOX@Fe-HMON-Tf NPs after biodegradation in FBS at 1d, 3d, 5d, 7d, and 14d. Scale bar = 100 nm. **Fig. S4**. Cell viability under different treatments. (a) Cytotoxicity of HMON-Tf NPs and Fe-HMON-Tf NPs after incubation with LO2 cells for 24h. ****P < 0.0001, two-tailed t test; mean ± SD. (b) Cytotoxicity of HMON-Tf NPs and Fe-HMON-Tf NPs after incubation with HepG2 cancer cells for 24 h. ****P < 0.0001, two-tailed t test; mean ± SD. (c) Cell viability of HepG2 cells treated with PBS, DOX, HMON-Tf NPs, Fe-HMON-Tf NPs, DOX@HMON-Tf NPs, DOX@Fe-HMON-PEG NPs or DOX@Fe-HMON-Tf NPs at different DOX dosage. **Fig. S5**. H2O2 levels in tumor tissue and changes in the intracellular lipid ROS levels after different treatments. (a) Western blot analysis on the expression of HIF-1α in HepG2 cells cultured in a normoxic environment and a hypoxic environment. (b) H2O2 levels of tumor tissues extracted from HepG2 tumor–bearing mice after the treatment with varied concentrations of DOX. n = 6; ****P < 0.0001, two-tailed t test; mean with SD. (c) Flow cytometric analysis and (d) CLSM observation on the intracellular lipoperoxide levels in HepG2 cells incubated with varied concentrations of DOX for 24h. Scale bar = 50um. (e) MDA levels in HepG2 cells incubated with PBS, DOX, HMON-Tf NPs, Fe-HMON-Tf NPs, DOX@HMON-Tf NPs, DOX@Fe-HMON-PEG NPs and DOX@Fe-HMON-Tf NPs for 24h. ***P < 0.001; ****P < 0.0001, two-tailed t test; mean ± SD. **Fig. S6**. Body weight changes of mice after different treatments and the corresponding in vivo biosafety evaluation. (a) Changes in the average body weight of tumor-bearing mice through the 21-day treatment period, which were recorded every 2 days. (b) Red blood cell (RBC); (c) white blood cell (WBC); (d) hemoglobin (HGB) and (e) blood platelet (PLT). (f) Blood levels of ALT, AST and ALP as liver function markers. (g) Blood urea nitrogen (BUN) and (h) creatinine (CREA) represent as kidney function markers. n = 6; mean ± SD. (i) Histological analysis of the major organs (lung, liver, spleen, kidney, heart) extracted from HepG2 -tumor bearing mice after the 21 days treatment with PBS, DOX, HMON-Tf NPs, Fe-HMON-Tf NPs, DOX@HMON-Tf NPs, DOX@Fe-HMON-PEG NPs and DOX@Fe-HMON-Tf NPs, for which the organ slices were processed for H&E staining. Scale bar = 100um.

## Data Availability

All data generated or analyzed during this study are included in this published. article and its additional file.
