# Supplementary material for: Self-amplification of oxidative stress with tumour microenvironment-activatable iron-doped nanoplatform for targeting hepatocellular carcinoma synergistic cascade therapy and diagnosis
Source: J Nanobiotechnology. 2021 Nov 8;19:361. doi: 10.1186/s12951-021-01102-0 (PMC8576982; doi:10.1186/s12951-021-01102-0)
Supplement: Supplementary file 1 — Additional file 1: Fig. S1. Nanoplatform characterisation. (a) Sizes and (b) ζ-potentials of HMON NPs, Fe-HMON NPs, Fe-HMON-PEG NPs, and Fe-HMON-Tf NPs. (c) Nitrogen sorption isotherms and (d) pore size distribution curves for Fe-HMON NPs. Fig. S2. Nanoplatform composition. (a) Coomassie Blue analysis of particles after transferrin modification with UV–vis spectroscopy. Fe-HMON-Tf NPs stained blue while Fe-HMON-PEG NPs stained green. (b) FTIR spectra of HMON NPs, Fe-HMON NPs, Fe-HMON-PEG NPs, and Fe-HMON-Tf NPs. Fig. S3. TEM images of DOX@Fe-HMON-Tf NPs after biodegradation in FBS at 1d, 3d, 5d, 7d, and 14d. Scale bar = 100 nm. Fig. S4. Cell viability under different treatments. (a) Cytotoxicity of HMON-Tf NPs and Fe-HMON-Tf NPs after incubation with LO2 cells for 24h. ****P < 0.0001, two-tailed t test; mean ± SD. (b) Cytotoxicity of HMON-Tf NPs and Fe-HMON-Tf NPs after incubation with HepG2 cancer cells for 24 h. ****P < 0.0001, two-tailed t test; mean ± SD. (c) Cell viability of HepG2 cells treated with PBS, DOX, HMON-Tf NPs, Fe-HMON-Tf NPs, DOX@HMON-Tf NPs, DOX@Fe-HMON-PEG NPs or DOX@Fe-HMON-Tf NPs at different DOX dosage. Fig. S5. H2O2 levels in tumor tissue and changes in the intracellular lipid ROS levels after different treatments. (a) Western blot analysis on the expression of HIF-1α in HepG2 cells cultured in a normoxic environment and a hypoxic environment. (b) H2O2 levels of tumor tissues extracted from HepG2 tumor–bearing mice after the treatment with varied concentrations of DOX. n = 6; ****P < 0.0001, two-tailed t test; mean with SD. (c) Flow cytometric analysis and (d) CLSM observation on the intracellular lipoperoxide levels in HepG2 cells incubated with varied concentrations of DOX for 24h. Scale bar = 50um. (e) MDA levels in HepG2 cells incubated with PBS, DOX, HMON-Tf NPs, Fe-HMON-Tf NPs, DOX@HMON-Tf NPs, DOX@Fe-HMON-PEG NPs and DOX@Fe-HMON-Tf NPs for 24h. ***P < 0.001; ****P < 0.0001, two-tailed t test; mean ± SD. Fig. S6. Body weight changes of [file 12951_2021_1102_MOESM1_ESM.pdf]

Supplementary Materials

Self-amplification of oxidative stress with tumour microenvironment-activatable iron-doped nanoplatform for targeting hepatocellular carcinoma synergistic cascade therapy and diagnosis

Qiao-Mei Zhou[1], Yuan-Fei Lu[1], Jia-Ping Zhou[1], Xiao-Yan Yang[1], Xiao-Jie Wang[1], Jie-Ni Yu[1], Yong-Zhong Du[2#], Ri-Sheng Yu[1#]

[1]Department of Radiology, Second Affiliated Hospital, School of Medicine, Zhejiang University, Hangzhou 310009, People's Republic of China

[2]Institute of Pharmaceutics, College of Pharmaceutical Sciences, Zhejiang University, Hangzhou 310058, People's Republic of China

#Corresponding author

Supplementary Materials Inventory

Fig. S1 Nanoplatform characterization.

Fig. S2 Nanoplatform composition.

Fig. S3 TEM images of DOX@Fe-HMON-Tf NPs after the biodegradation in FBS at varied time intervals.

Fig. S4 Cell viability under different treatments.

Fig. S5 H_2O_2 levels in tumor tissue and changes in the intracellular lipid ROS levels after different treatments.

Fig. S6 Body weight changes of mice after different treatments and the corresponding in vivo biosafety evaluation.

Supplementary Figure 1

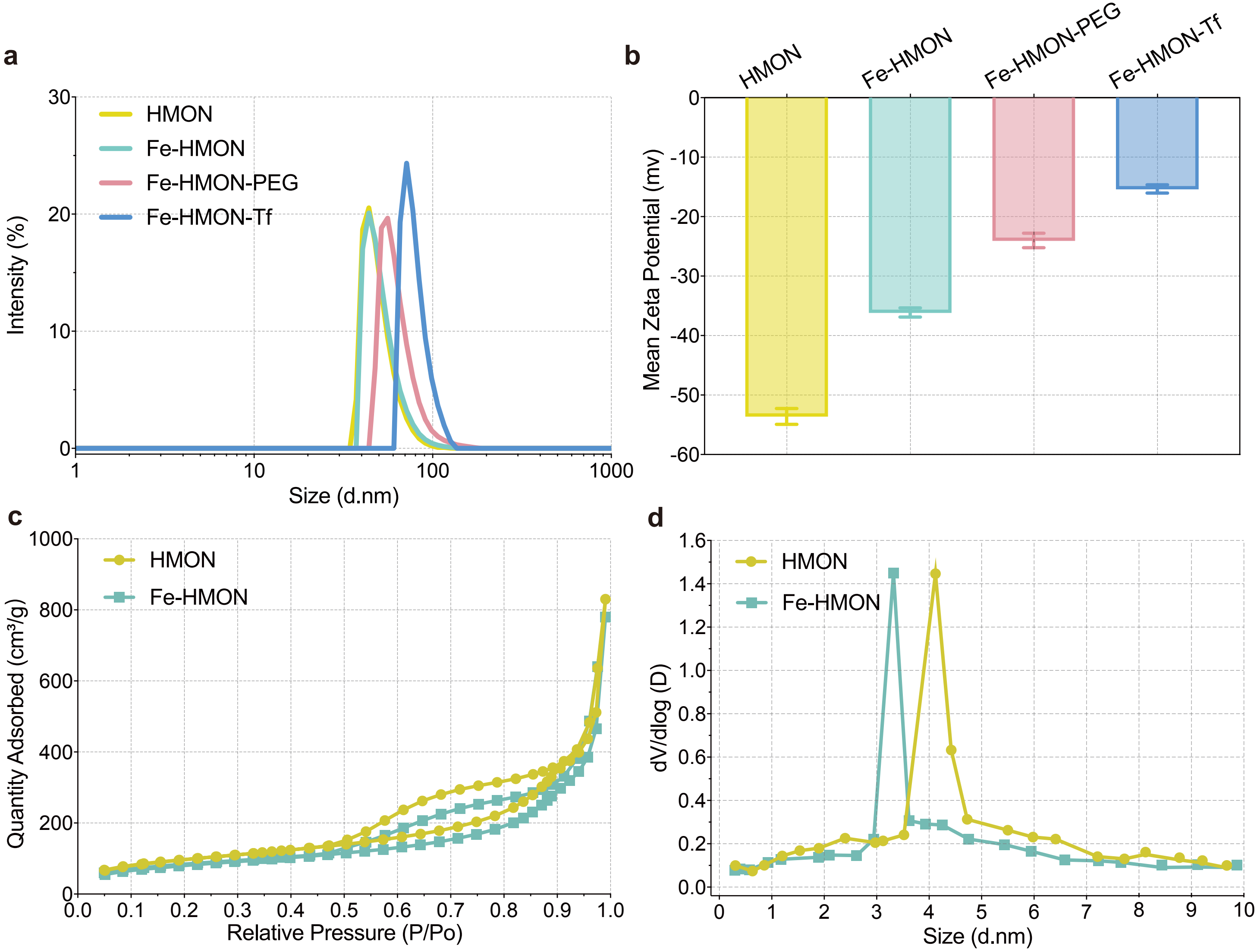

Fig. S1 Nanoplatform characterization. (**a**) Size and (**b**) ζ-potentials of HMON NPs, Fe-HMON NPs, Fe-HMON-PEG NPs, and Fe-HMON-Tf NPs. (**c**) Nitrogen sorption isotherms and (**d**) pore size distribution curves for Fe-HMON NPs.

Supplementary Figure 2

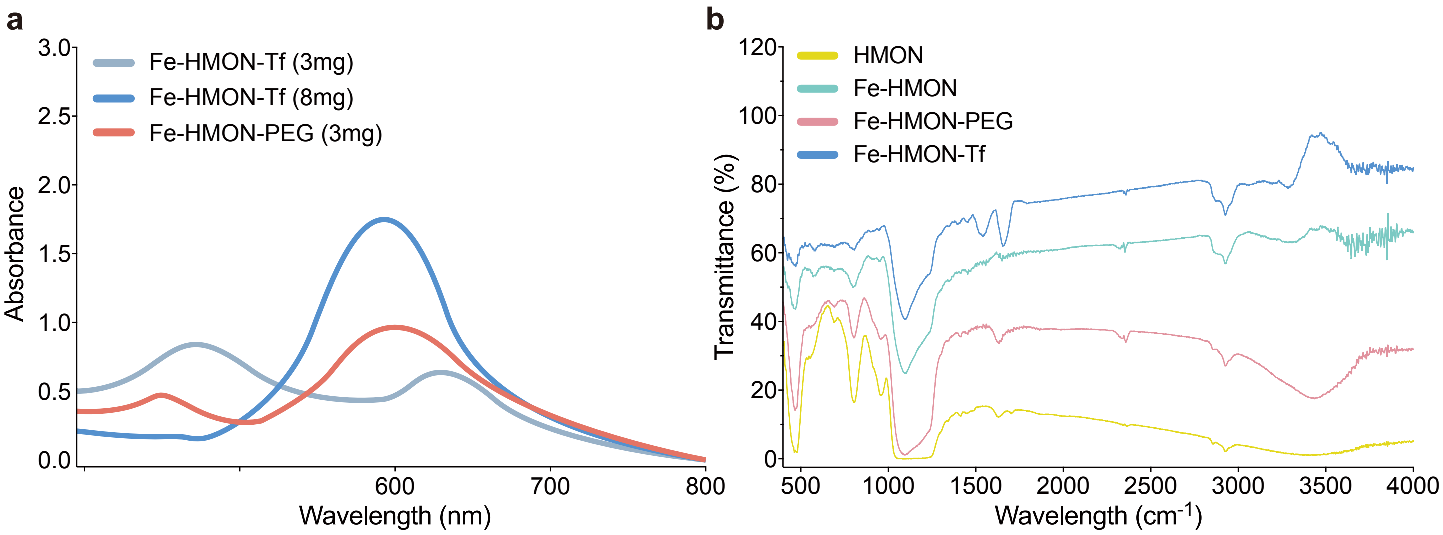

Fig. S2 Nanoplatform composition. (**a**) Coomassie Blue analysis of particles after transferrin modification with UV–vis spectroscopy. Fe-HMON-Tf NPs stained blue while Fe-HMON-PEG NPs stained green. (**b**) FTIR spectra of HMON NPs, Fe-HMON NPs, Fe-HMON-PEG NPs, and Fe-HMON-Tf NPs.

Supplementary Figure 3

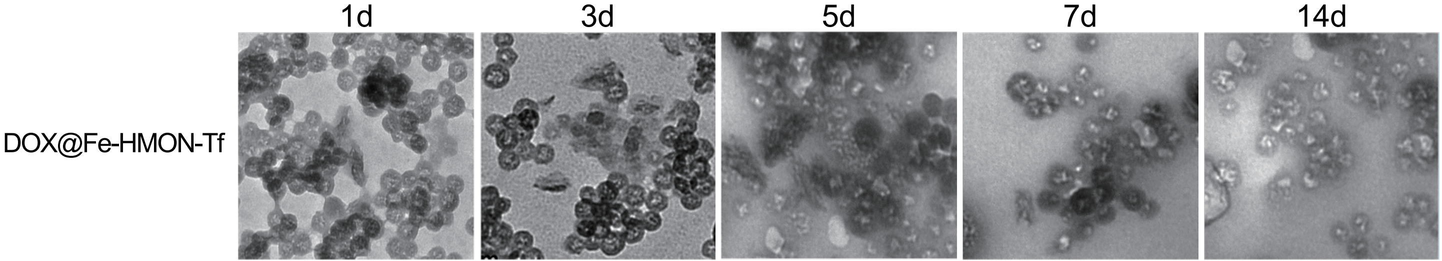

Fig. S3 TEM images of DOX@Fe-HMON-Tf NPs after the biodegradation in FBS at varied time intervals (1d, 3d, 5d, 7d and 14d).

Supplementary Figure 4

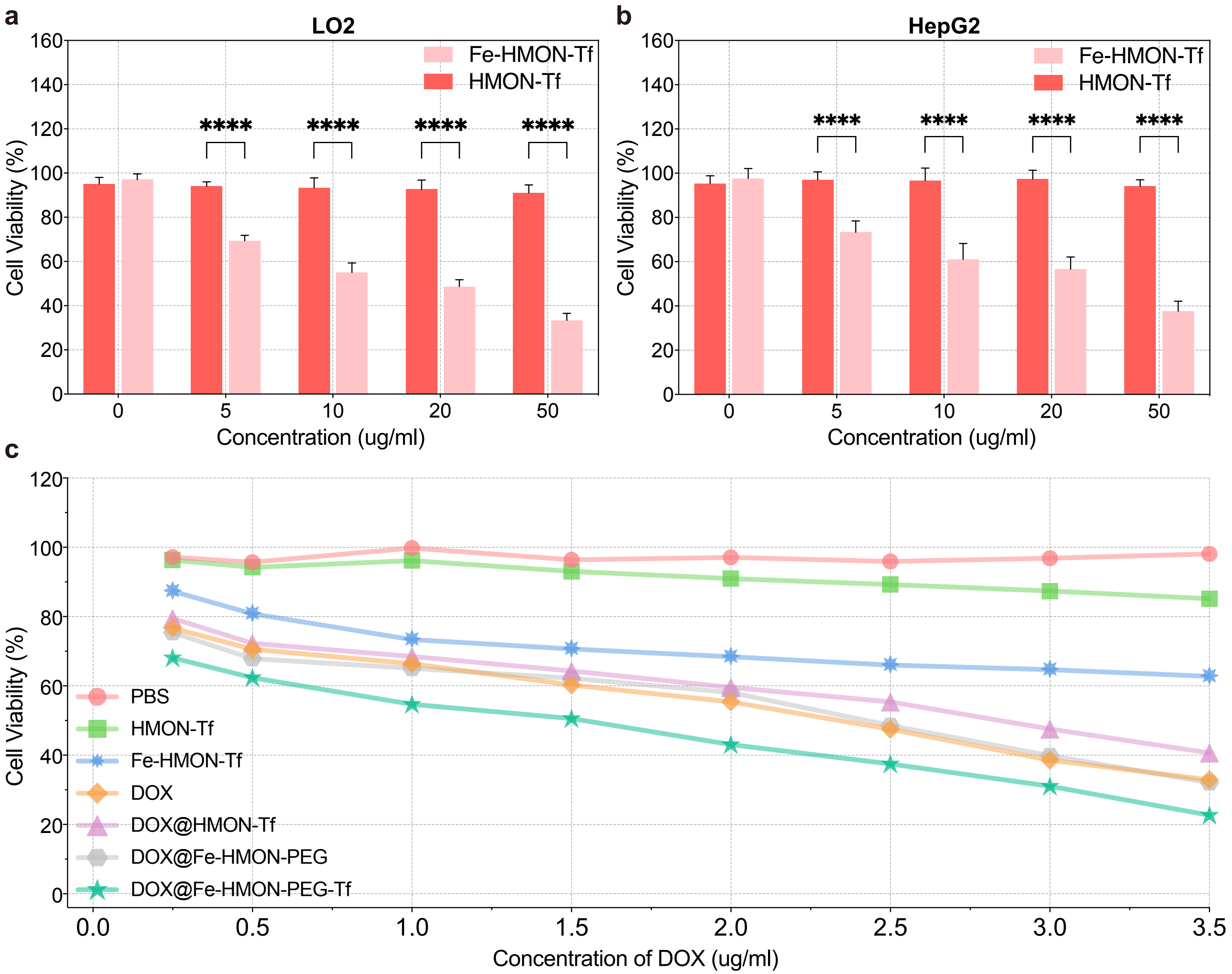

Fig. S4 Cell viability under different treatments. (**a**) Cytotoxicity of HMON-Tf NPs and Fe-HMON-Tf NPs after incubation with LO2 cells for 24h. ****$P < 0.0001$, two-tailed t test; mean ± SD. (**b**) Cytotoxicity of HMON-Tf NPs and Fe-HMON-Tf NPs after incubation with HepG2 cancer cells for 24h. ****$P < 0.0001$, two-tailed t test; mean ± SD. (**c**) Cell viability of HepG2 cells treated with PBS, DOX, HMON-Tf NPs, Fe-HMON-Tf NPs, DOX@HMON-Tf NPs, DOX@Fe-HMON-PEG NPs and DOX@Fe-HMON-Tf NPs at different DOX dosage.

Supplementary Figure 5

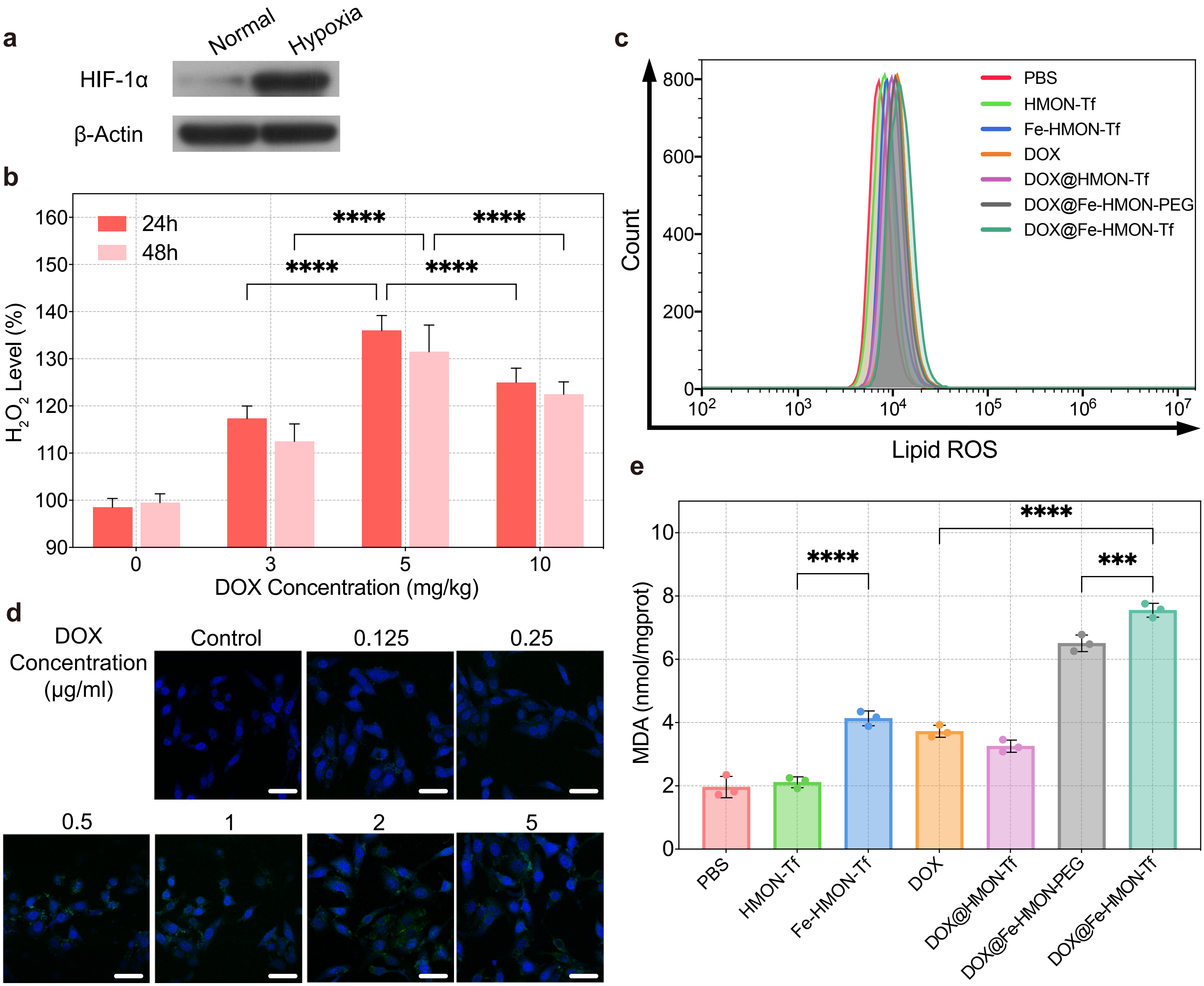

Fig. S5 H_2O_2 levels in tumor tissue and changes in the intracellular lipid ROS levels after different treatments. (**a**) Western blot analysis on the expression of HIF-1α in HepG2 cells cultured in a normoxic environment and a hypoxic environment. (**b**) H_2O_2 levels of tumor tissues extracted from HepG2 tumor–bearing mice after the treatment with varied concentrations of DOX. n=6; ****P < 0.0001, two-tailed t test; mean with SD. (**c**) Flow cytometric analysis and (**d**) CLSM observation on the intracellular lipoperoxide levels in HepG2 cells incubated with varied concentrations of DOX for 24h. Scale bar = 50um. (**e**) MDA levels in HepG2 cells incubated with PBS, DOX, HMON-Tf NPs, Fe-HMON-Tf NPs, DOX@HMON-Tf NPs, DOX@Fe-HMON-PEG NPs and DOX@Fe-HMON-Tf NPs for 24h. ***P < 0.001,****P < 0.0001, two-tailed t test; mean ± SD.

Supplementary Figure 6

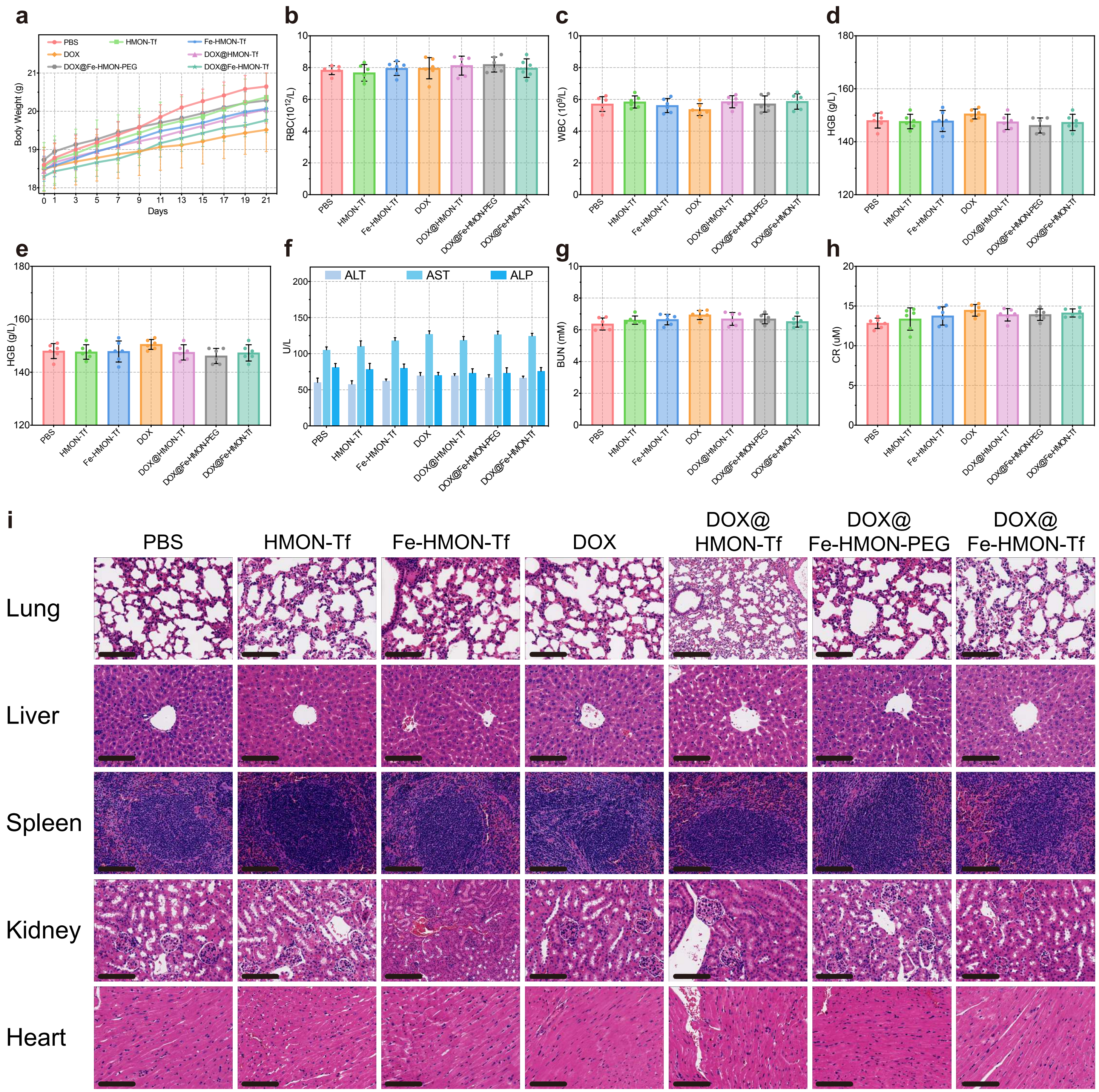

Fig. S6 Body weight changes of mice after different treatments and the corresponding in vivo biosafety evaluation. (**a**) Changes in the average body weight of tumor-bearing mice through the 21-day treatment period, which were recorded every 2 days. (**b**) Red blood cell (RBC); (**c**) white blood cell (WBC); (**d**) hemoglobin (HGB) and (**e**) blood platelet (PLT). (**f**) Blood levels of ALT, AST and ALP as liver function markers. (**g**) Blood urea nitrogen (BUN) and (**h**) creatinine (CREA) represent as kidney function markers. n = 6; mean ± SD. (**i**) Histological analysis of the major organs (lung, liver, spleen, kidney, heart) extracted from HepG2 -tumor bearing mice after the 21 days treatment with PBS, DOX, HMON-Tf NPs, Fe-HMON-Tf NPs, DOX@HMON-Tf NPs, DOX@Fe-HMON-PEG NPs and DOX-@Fe-HMON-Tf NPs, for which the organ slices were processed for H&E staining. Scale bar = 100um.
